# Bone marrow mesenchymal stem cells derived miRNA-130b enhances epithelial sodium channel by targeting PTEN

**DOI:** 10.1186/s12931-020-01595-7

**Published:** 2020-12-11

**Authors:** Honglei Zhang, Yan Ding, Yapeng Hou, Yanhong Liu, Zhiyu Zhou, Hongguang Nie

**Affiliations:** Department of Stem Cells and Regenerative Medicine, College of Basic Medical Science, China Medical University, Shenyang, 110122 People’s Republic of China

**Keywords:** Epithelial sodium channel, Bone marrow mesenchymal stem cells, miRNA-130b, Acute lung injury

## Abstract

**Aims:**

Acute lung injury (ALI) is a clinical syndrome with high morbidity and mortality, and severe pulmonary edema is one of the characteristics. Epithelial sodium channel (ENaC) located on the apical side of alveolar type 2 epithelial (AT2) cells is the primary rate limiting segment in alveolar fluid clearance. Many preclinical studies have revealed that mesenchymal stem cells (MSCs) based therapy has great therapeutic potential for ALI, while the role of ENaC in this process is rarely known.

**Methods:**

We studied the effects of bone marrow-derived MSCs (BMSCs) on the protein/mRNA expression and activity of ENaC in primary mouse AT2 and human H441 cells by co-culture with them, respectively. Moreover, the changes of miRNA-130b in AT2 cells were detected by qRT-PCR, and we studied the involvement of phosphatase and tensin homolog deleted on chromosome ten (PTEN) and the downstream PI3K/AKT pathway in the miRNA-130b regulation of ENaC.

**Results:**

Our results demonstrated that BMSCs could increase ENaC protein expression and function, as well as the expression level of miRNA-130b. The dual luciferase target gene assay verified that PTEN was one of the target genes of miR-130b, which showed adverse effects on the protein expression of α/γ-ENaC and PTEN in AT2 cells. Upregulating miR-130b and/or knocking down PTEN resulted in the increase of α/γ-ENaC protein level, and the protein expression of p-AKT/AKT was enhanced by miR-130b. Both α and γ-ENaC protein expressions were increased after AT2 cells were transfected with siPTEN, which could be reversed by the co-administration of PI3K/AKT inhibitor LY294002.

**Conclusion:**

In summary, miRNA-130b in BMSCs can enhance ENaC at least partially by targeting PTEN and activating PI3K/AKT pathway, which may provide a promising new direction for therapeutic strategy in ALI.

## Introduction

Acute lung injury (ALI) is a common clinical syndrome with high morbidity and mortality caused by sepsis, pneumonia, trauma, etc. [1, 2]. ALI and acute respiratory distress syndrome (ARDS) are characterized by an inflammatory response, alveolar edema, and hypoxemia. Approximately 40% of the ALI/ARDS patients are linked with viral and bacterial pneumonia [3, 4]. Lipopolysaccharide (LPS) is widely used to induce ALI models, which can attack pulmonary microvascular endothelial cells, and result in leakage of protein-rich edema fluid related with pulmonary endothelial cell injury, barrier dysfunction and inflammation [5]. Enhanced alveolar fluid clearance (AFC) can accelerate the clearance of edematous fluid accumulated in the pulmonary alveoli [6]. In the lungs, amiloride-sensitive epithelial sodium channel (ENaC) is the primary determinant of AFC, a driving force to eliminate edema fluid from alveolar spaces through the ion transport-dependent way [7, 8]. During the recovery of ALI, edematous fluid can be reabsorbed to the interstitium either by paracellular pathways or diffusion driven by an osmotic gradient that is established by active apical Na^+^ uptake, in part by the ENaC and Na^+^ transport through the Na^+^/K^+^-ATPase pumps [9]. There are mainly three subunits (α, β, and γ) to make up ENaC, which transports Na^+^ from apical to basolateral side of alveolar epithelial cells and regulates the transport of water [10, 11]. The α-subunit is required for Na^+^ conductance, while β- and γ-subunits are needed to enhance the channel activity [12].

Mesenchymal stem cells (MSCs) are pluripotent stem cells, which are featured by their ability to differentiate into multiple cell lines and exert antiproliferative, immunomodulatory and anti-inflammatory effects [13]. MSCs have the ability to regulate the immune response to tissue damage and promote repair in vivo, and have been suggested to benefit for a variety of pulmonary diseases (including ALI) [14]. In addition, MSCs can release a variety of cytokines and growth factors, signal lipids, exosomes and microRNAs (miRNAs, miRs), many of which may be involved in a variety of pulmonary diseases [15–17]. Related studies have shown that miR-130b in MSCs can regulate the inflammatory response, cell function and gene expression of various diseases [18–20], whereas its role in ALI and the relative mechanisms are seldom studied. Therefore, we chose miR-130b as our main research object and co-cultured with AT2 cells to explore its role in the regulation of bone marrow-derived MSCs (BMSCs) in LPS-induced ALI, aiming to provide a novel direction for therapeutic strategy in ALI.

Several bioinformatic websites such as TargetScan (http://www.targetscan.org/), PicTar (http://pictar.mdc-berlin.de/), and miRBase (http://www.mirbase.org/) are used to predict the targets of miR-130b, and a few targets are therefore found, such as phosphatase and tensin homolog deleted on chromosome ten (PTEN), IGF-1, PPAR-γ and CSF-1, etc. Moreover, PTEN has been reported to be the target of miR-130b and a negative regulator in the phosphatidylinositol 3-kinase (PI3K)/protein kinase B (AKT) signaling pathway that can up-regulate ENaC [21], which is the key step for the edematous fluid accumulation in ALI [22]. Thus we chose PTEN as the interested gene of miR-130b to study its role in pulmonary fluid transport accordingly. We speculate that BMSCs may affect ENaC through miR-130b targeting PTEN, and thus have a certain therapeutic effect on ALI.

## Materials and methods

### BMSC culture

All experimental protocols relating to C57 mice were performed according to the guidelines and regulations of Animal Care and Use Ethics Committee, and were approved by China Medical University (No. CMU2019088). The isolation and culture method of BMSCs has been described previously [23]. In brief, the femora were removed from C57 male mice and the medullary cavity of femora was washed with DMEM/F12 medium supplemented with 10% fetal bovine serum (FBS, Gibco, New York, NY, USA), 10 ng/ml recombinant mouse basic fibroblast growth factor (PeproTech, Rocky Hill, NJ, USA), 100 IU penicillin, and 100 μg/ml streptomycin, then the bone marrow was collected. After the cell suspension was mixed, the cells were cultured in 5% CO_2_–95% air at 37 °C for 24 h, and then the medium was changed to remove the non-adherent tissues and cells.

### Alveolar type 2 epithelial cell culture

Alveolar type 2 epithelial (AT2) cells were isolated and cultured as previously described [24]. Isolated lungs from newborn mice (within 24 h) were separated by lobes in cold PBS. The lung tissue was digested with trypsin and collagenase (Sigma, Saint Louis, MO, USA) for 30 min, respectively. Cells were filtrated and cultured in 5% CO_2_, 37 °C atmosphere in DMEM/F12 medium (containing 10% FBS, 100 IU penicillin, and 100 μg/ml streptomycin) for 45 min. Unattached cells were collected and the above culture process was repeated 4 times to remove lung fibroblast cells. Then, the cell suspension was transferred to the culture dish coated IgG and incubated for 30 min to remove lymphocytes, macrophages, and neutrophils. Finally, unattached cells were adjusted to 2–3 × 10^6^/ml and the medium was changed after 72 h for the first time and then changed every other day.

### Co-culture of BMSCs and AT2 cells

BMSCs were passaged after 80% confluence and the cells of the 2nd and 3rd passages were used to culture in 24-mm diameter Transwell inserts. After 24 h, the BMSC inserts were transferred to 6-well plates with co-cultured AT2 cells at the bottom, both the inserts and lower culture wells were washed three times with PBS, and then the medium was switched to DMEM/F12 without FBS for 24 h.

### ELISA measurement

The concentration of interleukin-8 (IL-8) in the mouse AT2 cell supernatant was measured by ELISA kit (EMC104, NeoBioScience, Shenzhen, China), and the operation process was strictly in accordance with the manufacturing instructions. In general, the antigens were added to the primary antibody and then overlaid with the secondary antibody to form a superimposed pattern. Then after TMB colored, we measured the OD at 450 nm with microplate.

### Ussing chamber assay

H441 cells obtained from the American Type Culture Collection were seeded onto 6.5-mm diameter mouse tail collagen I pre-coated Transwell inserts (~ 6 × 10^6^ cells/cm^2^), and cultured with RPMI-1640 medium containing 10% FBS, 100 IU penicillin, 100 μg/ml streptomycin, 10 μg/ml insulin, and 50 nM dexamethasone in 5% CO_2_–95% air at 37 °C. After 24 h, the medium and nonadherent cells in the apical compartment were removed to adapt the cells to air–liquid interface culture, and the medium in the basolateral compartment was replaced. Trans-epithelial electrical resistance was monitored by an epithelial volt-ohm-meter (WPI, Sarasota, FL, USA), and cell-growing inserts with a resistance > 400 Ω cm^2^ were used.

Measurements of trans-epithelial electrical resistance and short-circuit current (*Isc*) were performed as previously described [25]. Briefly, H441 monolayers were mounted in ussing chambers (Physiologic Instruments, San Diego, CA, USA) and bathed on both sides with solutions containing (in mM) 120 NaCl, 25 NaHCO_3_, 3.3 KH_2_PO_4_, 0.83 K_2_HPO_4_, 1.2 CaCl_2_, 1.2 MgCl_2_, 10 HEPES, and 10 mannitol (apical compartment)/10 glucose (basolateral compartment). The trans-epithelial *Isc* level was measured with 3 M KCl, 4% agarose salt bridges placed 3 mm on either side of the membrane, which were connected on either side to Ag–AgCl electrodes. Both sides with the previously mentioned bath solution (pH 7.4) were bubbled continuously with 95% O_2_–5% CO_2_ gas mixture and the temperature was set as 37 °C. H441 monolayers were short circuited to 0 mV, and *Isc* level was measured with an epithelial voltage clamp. A 10 mV pulse of 1 s duration was imposed every 10 s to monitor trans-epithelial electrical resistance. When the *Isc* was stable, 100 μM amiloride was pipetted into the apical side. Data were collected using the Acquire and Analyse program version 2.3.

### Cell transfection

PTEN-siRNA (siPTEN), miR-130b mimic (Mimic), miR-130b inhibitor (Inhibitor), negative control (NC, the negative control of miR-130b mimic or PTEN-siRNA), inhibitor NC (the negative control of miR-130b inhibitor), Cy3-miR-130b, and siRNA-mate were purchased from GenePharma (Shanghai, China). The final concentration of miR-130b mimic, miR-130b inhibitor, and PTEN-siRNA were 30 nM, 60 nM, and 200 nM, respectively. All transfection reagents were removed after 6 h and cells were used 72 h after transfection, and miR-130b labelled with Cy3 was used for fluorescence detection.

### Western blot assays

The cell lysates were separated by SDS-PAGE (10% polyacrylamide gels) and transferred onto PVDF membrane. Membrane blockade was blocked with 5% BSA for 1 h at room temperature, and then incubated with diluted primary antibodies overnight: α-ENaC (1:2000, PA1-920A, Thermo Fisher, Waltham, MA, USA), γ-ENaC (1:2000, ab3468, Abcam, Cambridge, MA, USA), PTEN (1:1000, 9552S, Cell signaling, Danvers, MA, USA), pAKT (1:1000, ab38449, Abcam, Cambridge, MA, USA), AKT (1:1000, ab8933, Abcam, Cambridge, MA, USA), and β-actin (1:1000, sc-47778, Santa Cruz Biotechnology, Santa Cruz, CA, USA). The membranes were washed three times and incubated with HRP conjugated goat-anti-rabbit or goat-anti-mouse secondary antibody (1:5000, ZSGB-BIO, Beijing, China) at room temperature for 1 h. The protein bands were visualized using ECL kit on a Tanon-5200 chemiluminescence detection system (Tanon, Shanghai, China), and the intensity of each specific band was quantified with Image J program.

### Quantitative real-time PCR

Total RNA was isolated using TRIzol reagent (Invitrogen, Carlsbad, CA, USA) and quantified by NanoDrop 2000C spectrophotometer (Thermo, Wilmington, DE, USA). In brief, total RNA and miRNA were synthesized into cDNA using PrimeScript RT reagent kit with gDNA Eraser and Mir-X miRNA First-Strand Synthsis Kit (TaKaRa, Kusatsu, Shiga, Japan). Quantitative real-time PCR (qRT-PCR) was then applied using SYBR Premix Ex Taq II (TaKaRa, Kusatsu, Shiga, Japan) in the ABI 7500 qRT-PCR System with the following primers: α-ENaC forward (5′-AAC AAA TCG GACTGC TTC TAC-3′) and reverse (5′-AGC CAC CAT CAT CCA TAA A-3′), β-ENaC forward (5′-GGG ACC AAA GCA CCA AT-3′) and reverse (5′-CAG ACG CAG GGA GTC ATAG-3′), γ-ENaC forward (5′-GCACCG TTC GCC ACC TTC TA-3′) and reverse (5′-AGG TCA CCA GCA GCT CCT CA-3′), and GAPDH forward (5′-AGA AGG CTG GGG CTC ATT TG-3′) and reverse (5′-AGG GGC CAT CCA CAG TCT TC-3′). Relative expression of mRNA/miRNA was calculated using the 2^−Δ(ΔCT)^ method, and GAPDH/U6 was used as a reference.

### Dual luciferase reporter gene assay

The dual luciferase reporter gene detects the regulation of genes by reflecting the amount of luciferase expression. It detects the fluorescence intensity of fluorescein substrate after transfecting cells with a reporter plasmid. H441 cells were cultured in a six-well plate, and fused to 60–70%. The constructed PTEN-3′UTR wild-type and mutant recombinant plasmids (GenePharma) were transfected into H441 cells with miR-130b mimic or miR-130b mimic negative control, respectively. After 48 h, luciferase activity was measured using the Dual Luciferase Reporter Assay Kit (Vazyme), according to the manufacturer’s instructions.

### Statistical analysis

Data were expressed as the mean ± SE. We evaluated the power of sample size first to meet *P* < 0.05. Normality and homoscedasticity test was done by Levene and Shapiro–Wilk test before applying parametric tests. For comparison of two groups, we used Student’s two-tailed t-test; for comparison of multiple groups, we performed one-way analysis of variance (ANOVA) followed by Bonferroni’s test for all the groups of the experiment. When the data did not pass the normality or homoscedasticity test, we used a non-parametric t-test (Mann–Whitney U test). Statistical analysis was performed with Origin 8.0.

## Results

### BMSCs increase α/γ-ENaC protein and mRNA expression

**Fig. 1:**
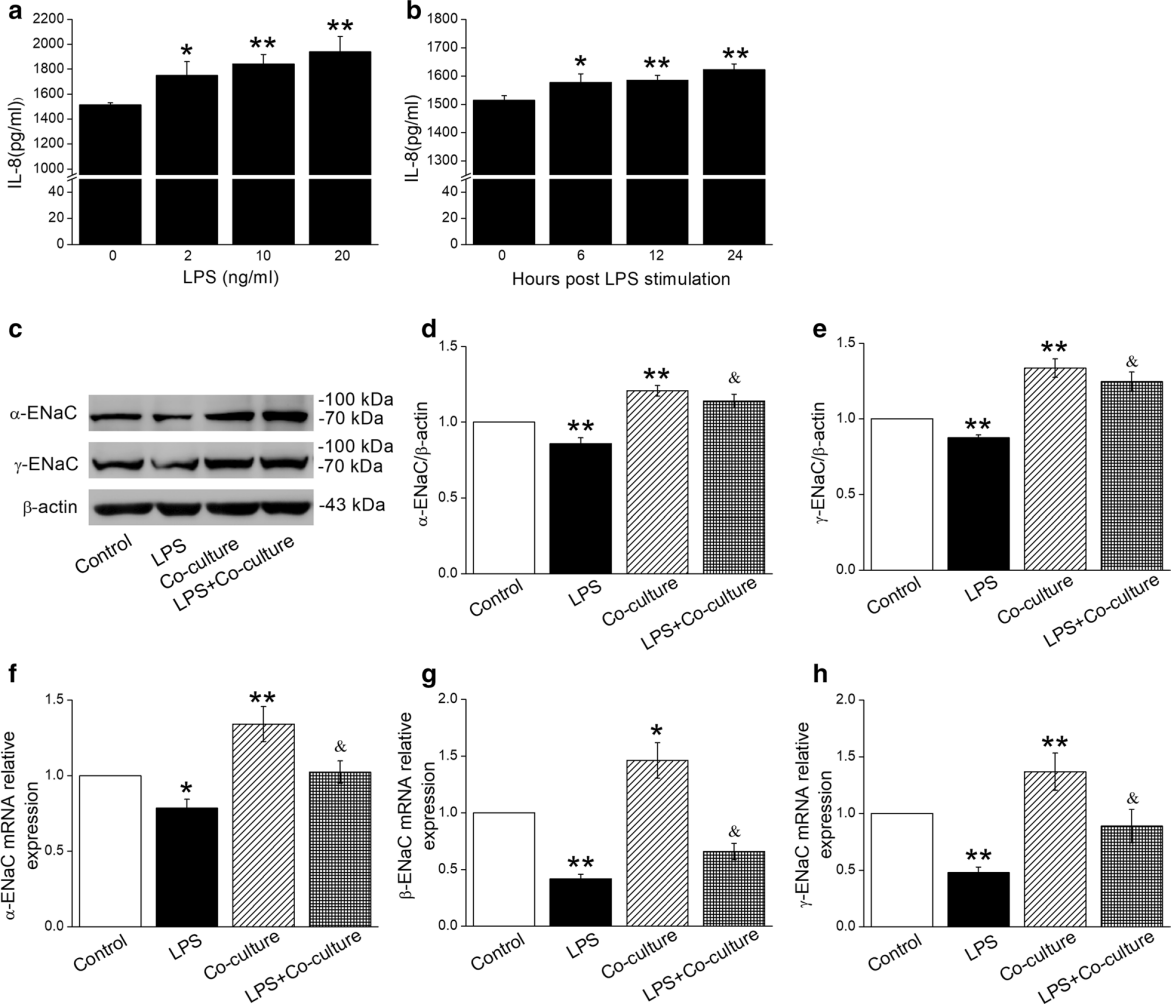
LPS treatment enhances IL-8 levels and BMSCs increase protein and transcription level of ENaC in AT2 cells. **a**, **b** IL-8 levels were measured by ELISA kit after LPS treatment of different dosage and time in AT2 cells. **c** Representative Western blot bands of α- and γ-ENaC protein expression in AT2 cells treated with LPS for 12 h and/or co-cultured with BMSCs for 24 h. Blots for β-actin were used as internal controls. **d**, **e** Graphical representation of data obtained from Western blot and quantified through gray analysis (α- or γ-ENaC/β-actin). **f**–**h** qRT-PCR results for ENaC mRNAs. Relative level of ENaC mRNA were calculated as α-, β- or γ-ENaC/GAPDH ratios. *P < 0.05, **P < 0.01, compared with control; ^&^*P* < 0.05, compared with LPS, n = 5–6

Inflammatory cytokines are crucial factors in measuring LPS-induced ALI [26]. The LPS-induced IL-8 production in mouse AT2 cells became higher with the increase of dosage and time duration after LPS administration, and we chose 10 ng/ml LPS with 12 h duration for the future study (Fig. 1a, b). ENaC is mainly composed of α, β and γ subunits, that are all indispensable for efficient AFC [27]. To investigate the effect of BMSCs on the expression of ENaC in AT2 cells, we applied Western blot and qRT-PCR assays in normal or LPS-treated AT2 cells co-cultured with BMSCs. The data of the other groups in the same experiment were compared with that of the Control group (divided by the Control value). Firstly, BMSCs could increase the expression of α/γ-ENaC protein in both normal and LPS-treated AT2 cells (Fig. 1c–e), indicating the beneficial effects of BMSCs in ALI. Due to the lack of suitable antibody for the Western blot assay, we didn’t detect the β-ENaC expression. Furthermore, the results of qRT-PCR verified that BMSCs enhanced protein expression of ENaC was due to the higher transcription level in AT2 cells after LPS administration (Fig. 1f–h). BMSCs can enhance both the protein and mRNA expression of ENaC, supporting our hypothesis that ENaC is involved in the protective effects of BMSCs in ALI.

### BMSCs enhance amiloride-sensitive Isc in H441 monolayers

**Fig. 2:**
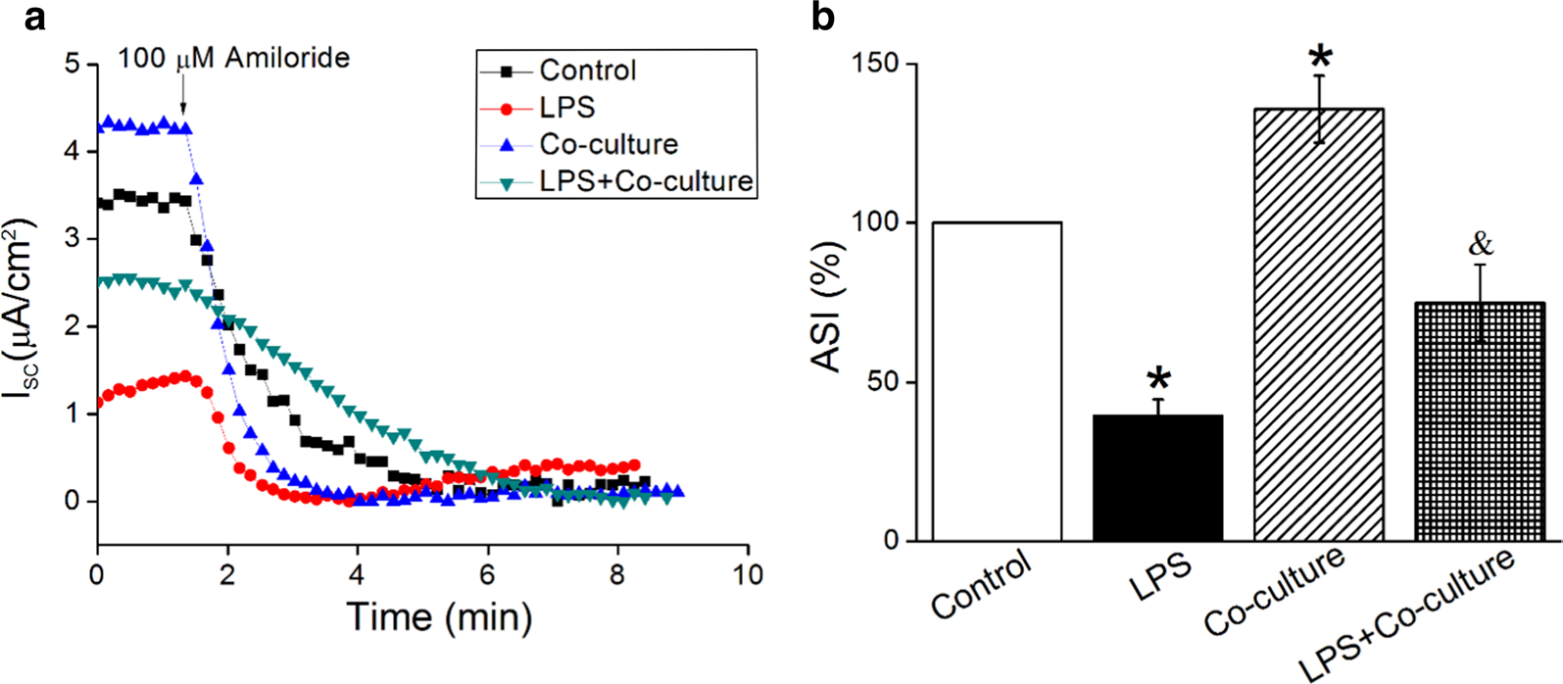
*Isc* level in H441 monolayers is enhanced by BMSCs. **a** Representative *Isc* traces after H441 monolayers were treated with LPS for 12 h and/or co-cultured with BMSCs for 24 h, then amiloride (100 μM) was applied. **b** Statistic ASI in H441 monolayers. ASI is defined as the total current value minus the plateau current value after adding amiloride, and the initial ASI is set to 100%. *P < 0.05, compared with control; ^&^*P* < 0.05, compared with LPS, n = 4

Human bronchoalveolar epithelial-derived club (H441) cells have been extensively applied in studying the function of ENaC in the lung, and ENaC properties of H441 are similar to those of primary AT2 cells [28], which could hardly grow into monolayers. To further confirm the regulation of BMSCs on ENaC activity, we measured *Isc* in confluent H441 monolayers. As shown in Fig. 2a, the *Isc* at time 0 was the total current measured in H441 monolayers, which reflected the activities of both apical and basolateral channels/transporters. The remaining *Isc* after adding amiloride was the fraction that was amiloride-resistant, while the amiloride-sensitive *Isc* (ASI, %) reflecting the ENaC activity was defined as the difference between the total current and the amiloride-resistant current, then divided by the initial ASI (× 100). The reason why BMSCs caused higher *Isc* at time 0 illustrated that BMSCs could not only increase ASI which was mainly mediated by ENaC, but also the total current. BMSCs significantly rescued the ASI reduction induced by LPS for 12 h. The above data further indicate that BMSCs can promote the ion transport of lung epithelium and possible edema fluid absorption, through increasing ENaC activity both under normally physiological and LPS-induced pathological conditions.

### MiR-130b in BMSCs increase α/γ-ENaC protein expression

**Fig. 3:**
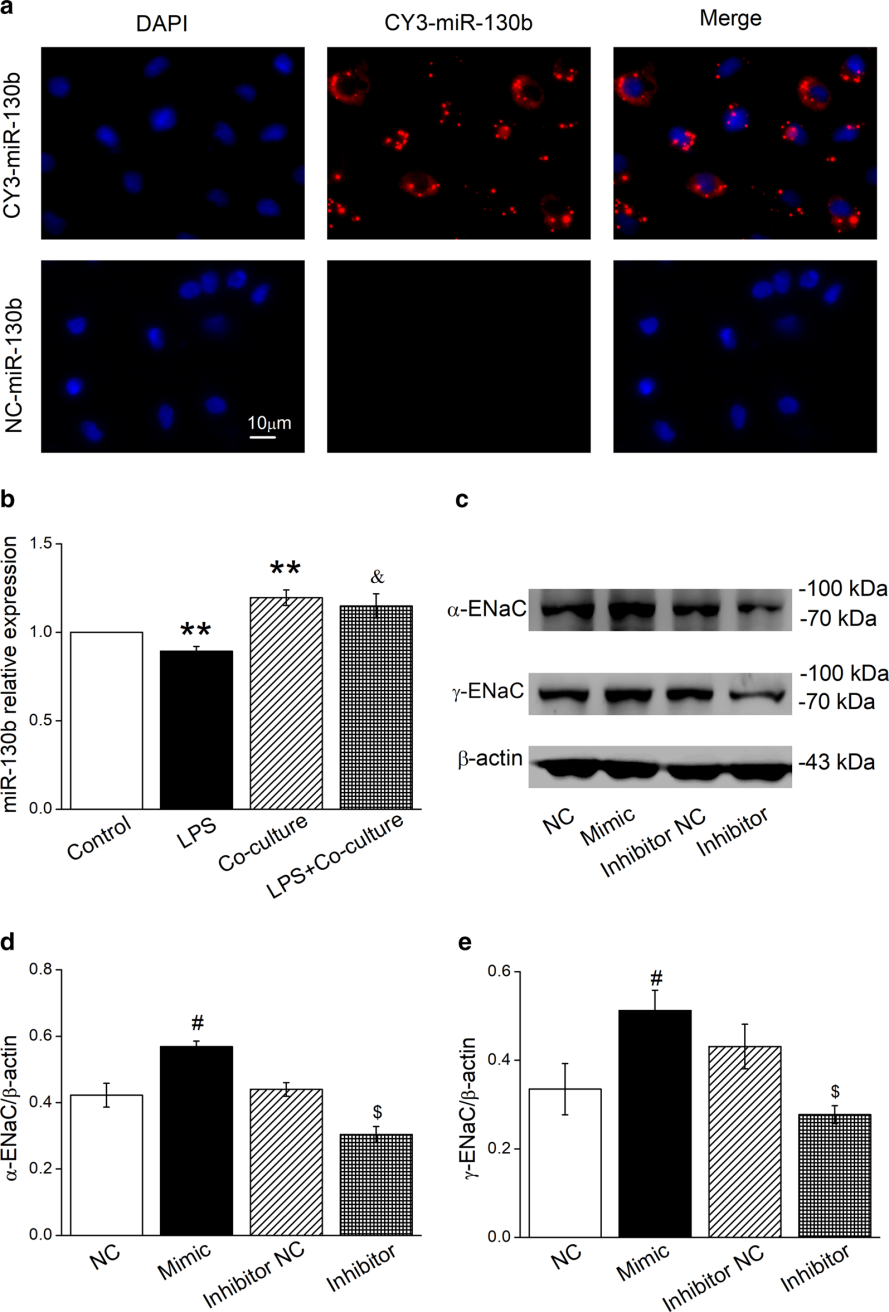
MiR-130b can be transferred into AT2 cells co-cultured with BMSCs and increases protein level α/γ-ENaC in AT2 cells. **a** Transfected miR-130b labelled with Cy3 was localized in AT2 cells co-cultured with BMSCs for 24 h. BMSCs were transiently transfected with Cy3-miR-130b after which they were co-cultured with mouse AT2 cells and then fixed. Nuclei were counterstained by 4,6-diamidino-2-phenylindole (DAPI, blue; left panel), and the representative staining images were shown in CY3-miR-130b positive AT2 cells (red; middle panel). The merged images were seen (right panel). Scale bar, 10 μm. **b** The result of real-time PCR assays shows miR-130b level in AT2 cells treated with LPS for 12 h and/or co-cultured with BMSCs for 24 h. The relative level of miR-130b were calculated as miR-130b/U6 ratio. **c** Representative Western blot bands of α- and γ-ENaC protein expression in AT2 cells transfected with miR-130b mimic/inhibitor for 72 h. Blots for β-actin were used as internal controls. **d**, **e** Graphical representation of data obtained from Western blot assays for α- and γ-ENaC subunits. Bands were quantified using gray analysis (α-ENaC/β-actin and γ-ENaC/β-actin). **P < 0.01, compared with control; ^&^*P* < 0.05, compared with LPS; ^#^*P* < 0.05, compared with miR-130b mimic negative control (NC); ^$^*P* < 0.05, compared with miR-130b inhibitor negative control (Inhibitor NC), n = 4–6

Growing evidence has indicated that miRNAs secreted by MSCs played a role in the treatment of pulmonary diseases [29]. To provide a direct evidence that miR-130b in BMSCs can be transferred into epithelial cells they are co-cultured with, we delivered the fluorescently labeled Cy3-miR-130b to the BMSCs and observed its distribution in co-cultured AT2 cells. As shown in Fig. 3a, the miRNA labelled with Cy3 was found in the cytoplasm of AT2 cells co-cultured with BMSCs. Compared with Control group that expressed miR-130b in normal AT2 cells, exposure to LPS caused a significant decrease of miR-130b (Fig. 3b). Conversely, BMSCs rescued the miR-130b reduction in LPS-treated cells. This indicates that miR-130b is likely to be a key factor for BMSCs to enhance ENaC.

In order to verify whether miR-130b affects ENaC, AT2 cells were transfected with miR-130b mimic (Mimic) or miR-130b inhibitor (Inhibitor), respectively. The effect of miR-130b on ENaC was examined by Western blot analysis. As expected, miR-130b inhibitor abrogated the promotion effect of miR-130b on α/γ-ENaC protein level Fig. 3c–e, supporting that miR-130b may be involved in the BMSCs enhanced ENaC expression.

### MiR-130b and BMSCs reduce PTEN protein expression

**Fig. 4:**
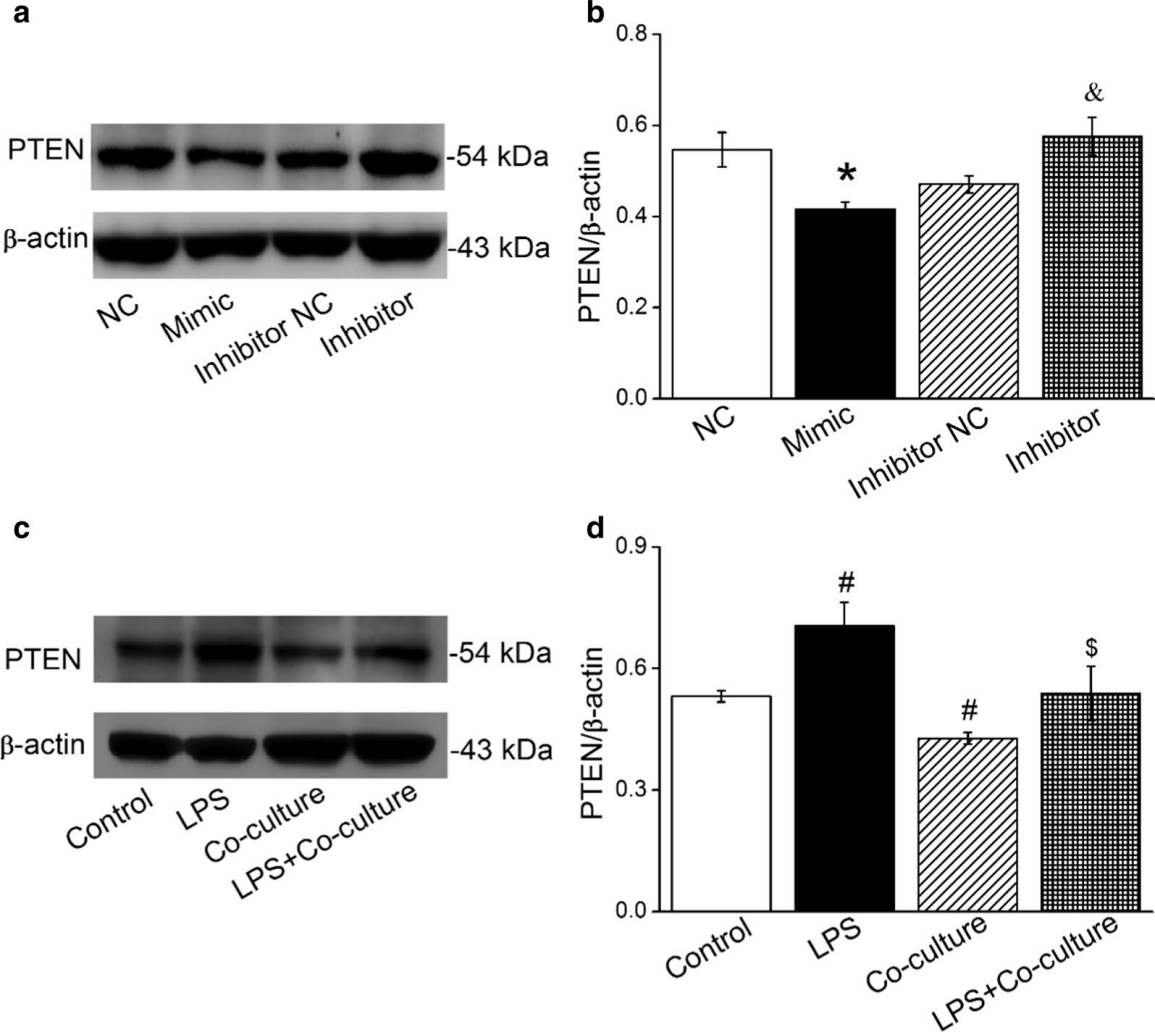
MiR-130b and BMSCs decrease protein level PTEN in AT2 cells. **a**, **c** Representative Western blot bands of PTEN protein expression in AT2 cells transfected with miR-130b mimic/inhibitor or co-cultured with BMSCs, respectively. **b**, **d** Graphical representation of data obtained from Western blot assays for PTEN. Bands were quantified using gray analysis (PTEN/β-actin). *P < 0.05, compared with miR-130b mimic negative control (NC); ^&^*P* < 0.05, compared with miR-130b inhibitor negative control (Inhibitor NC); ^#^*P* < 0.05, compared with control; ^$^*P* < 0.05, compared with LPS, n = 4–5

Potential miR-130b targets were predicted using in silico approaches, and according to the bioinformatic website prediction. Additional file 1: Figure S1 showed one of the screening results from the websites, which indicated that PTEN could be a potent target for miR-130b according to the values shown on the left of this screenshot. Besides, the related study revealed that miR-130b in BMSCs could target PTEN, which inversely correlated with miR-130b expression [30]. To test this hypothesis, AT2 cells were transfected with miR-130b mimic or inhibitor, respectively. As expected, transfection of miR-130b mimic resulted in a significant decrease of PTEN expression compared with the miR-130b mimic negative control (NC) group, while the inhibition of miR-130b showed an augment compared with the miR-130b inhibitor negative control (Inhibitor NC) group (Fig. 4a, b).

Based on the higher expression of miR-130b in AT2 cells after co-culture with BMSCs and the above adverse effects of miR-130b and PTEN, we hypothesized that BMSCs may downregulate PTEN in LPS-treated AT2 cells. To confirm this hypothesis, we applied Western blot to examine the effect of BMSCs on PTEN protein expression level. As expectedly, BMSCs suppressed the increase of PTEN in AT2 cells after LPS administration (Fig. 4c, d), supporting that PTEN is at least one of the targets in BMSCs regulating ENaC expression.

### BMSCs reduce PTEN and increase α/γ-ENaC protein expression via miR-130b

**Fig. 5:**
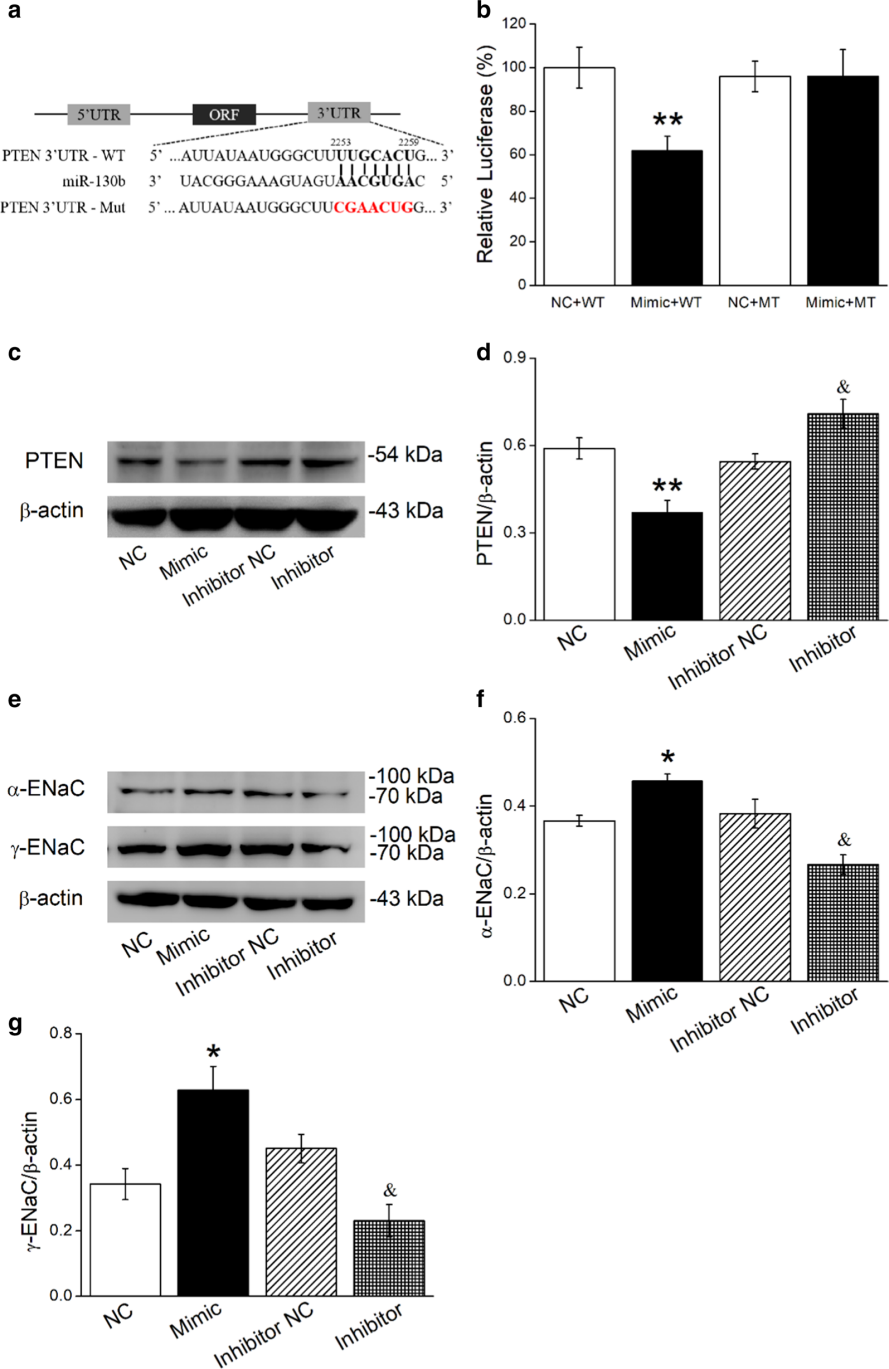
MiR-130b directly targets PTEN and BMSCs transfected with miR-130b mimic/miR-130b inhibitor affect protein expressions of PTEN and α/γ-ENaC in AT2 cells. **a** The potential binding sites for miR-130b on the 3′-UTRs of PTEN. **b** Dual luciferase assay for miR-130b binding with PTEN in H441 cells. H441 cells were co-transfected with miR-130b mimic negative control (NC) or miR-130b mimic (Mimic) together with pmirGLO-PTEN (WT or MT) for 24 h. **c**, **e** Representative Western blot bands of PTEN and α/γ-ENaC protein expression in AT2 cells co-cultured with BMSCs that were transfected with miR-130b mimic/inhibitor. **d**, **f** and** g** Graphical representation of data obtained from Western blot assays for PTEN and α/γ-ENaC. Bands were quantified using gray analysis (PTEN/β-actin, α-ENaC/β-actin and γ-ENaC/β-actin). *P < 0.05, **P < 0.01, compared with miR-130b mimic negative control or plus wild-type pmirGLO-PTEN (NC or NC + WT); ^&^*P* < 0.05, compared with miR-130b inhibitor negative control (Inhibitor NC), n = 4

Based on the above results, we speculate that miR-130b may exert the intermediate effect between BMSCs and PTEN/ENaC. To confirm the possibly direct binding of 130b and PTEN, a dual luciferase target gene assay was conducted, and H441 cells were co-transfected with the miR-130b mimic negative control (NC) or miR-130b mimic (Mimic), respectively. Meanwhile, wild-type (WT) and mutant reporter gene vectors (MT) for PTEN were constructed according to the potential binding sites for miR-130b on the 3′-UTRs of PTEN (Fig. 5a). We found that the expression of pmirGLO-PTEN-WT (Mimic + WT) relative luciferase activity was reduced significantly by miR-130b, while the expression of pmirGLO-PTEN-MT (Mimic + MT) was not suppressed (Fig. 5b). The above data verified that PTEN was one of the target genes of miR-130b, which could upregulate ENaC expression by binding to the 3′-UTR of PTEN.

Next miR-130b mimic or miR-130b inhibitor was transfected into BMSCs, respectively, which were then co-cultured with the AT2 cells. After co-culture with BMSCs overexpressing miR-130b, PTEN protein expression in AT2 cells decreased significantly, while BMSCs transfected with miR-130b inhibitor increased PTEN protein expression (Fig. 5c, d). Furthermore, miR-130b overexpression in BMSCs increased α/γ-ENaC protein expression in AT2 cells accordingly (Fig. 5e–g), indicating that BMSCs play a vital role in ENaC regulation through miR-130b, which may be achieved by targeting PTEN.

### PTEN gene knockdown enhanced the protein expression of α/γ-ENaC in AT2 cells

**Fig. 6:**
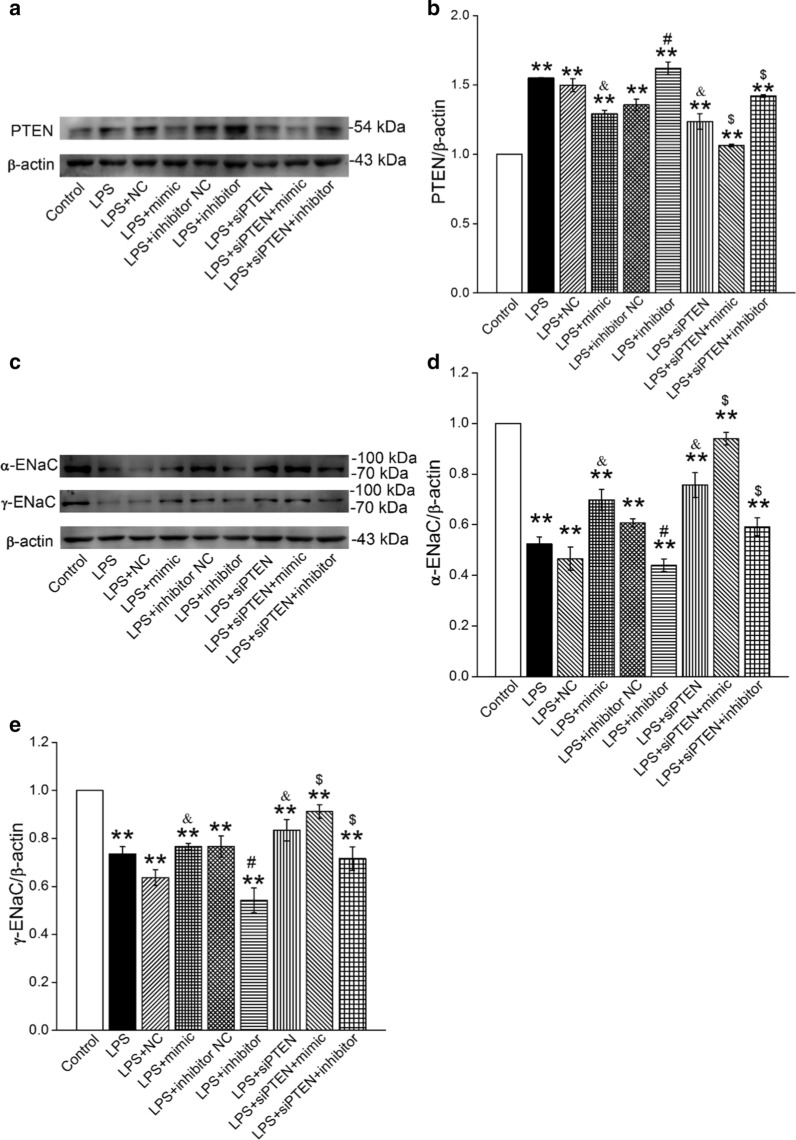
*PTEN* gene knockdown enhanced the protein expression of α/γ-ENaC in AT2 cells. **a**, **c** Representative Western blot bands of PTEN and α/γ-ENaC protein expression in LPS-treated AT2 cells transfected with miR-130b mimic/inhibitor or siPTEN. **b**, **d** and **e** Graphical representation of data obtained from Western blot assays for PTEN and α/γ-ENaC. Bands were quantified using gray analysis (PTEN/β-actin, α-ENaC/β-actin and γ-ENaC/β-actin). **P < 0.01, compared with control; ^&^*P* < 0.05, compared with LPS + miR-130b mimic negative control (NC); ^#^*P* < 0.05, compared with LPS + miR-130b inhibitor negative control (Inhibitor NC); ^$^*P* < 0.05, compared with LPS + siPTEN, n = 4–6

In order to further verify whether miR-130b exerts a protective effect on LPS-reduced ENaC by targeting PTEN, we knocked down the PTEN gene by siRNA to find out the role of PTEN in miR-130b regulation of ENaC. The siRNA transfection efficiency was verified by the Western blot analysis (Additional file 2: Figure S2). The protein expression of PTEN in the LPS-treated AT2 cells was reduced through miR-130b transfection, whereas inhibition of miR-130b displayed a contrasting effect (Fig. 6a, b). Meanwhile, both upregulating miR-130b and knocking down PTEN resulted in the increase of α/γ-ENaC protein level. In addition, when PTEN-siRNA and miR-130b mimic were co-transfected into LPS-treated AT2 cells, the expression of α/γ-ENaC protein were higher than that of PTEN-siRNA transfected alone, showing the synergistic effects (Fig. 6c–e). These results indicate that miR-130b can enhance the expression of α/γ-ENaC protein in LPS-treated AT2 cells, and PTEN participates in the regulation process of miR-130b on ENaC, which may be involved in BMSC treatment of ALI.

### MiR-130b regulates α/γ-ENaC through PTEN/PI3K/AKT pathway in AT2 cells

**Fig. 7:**
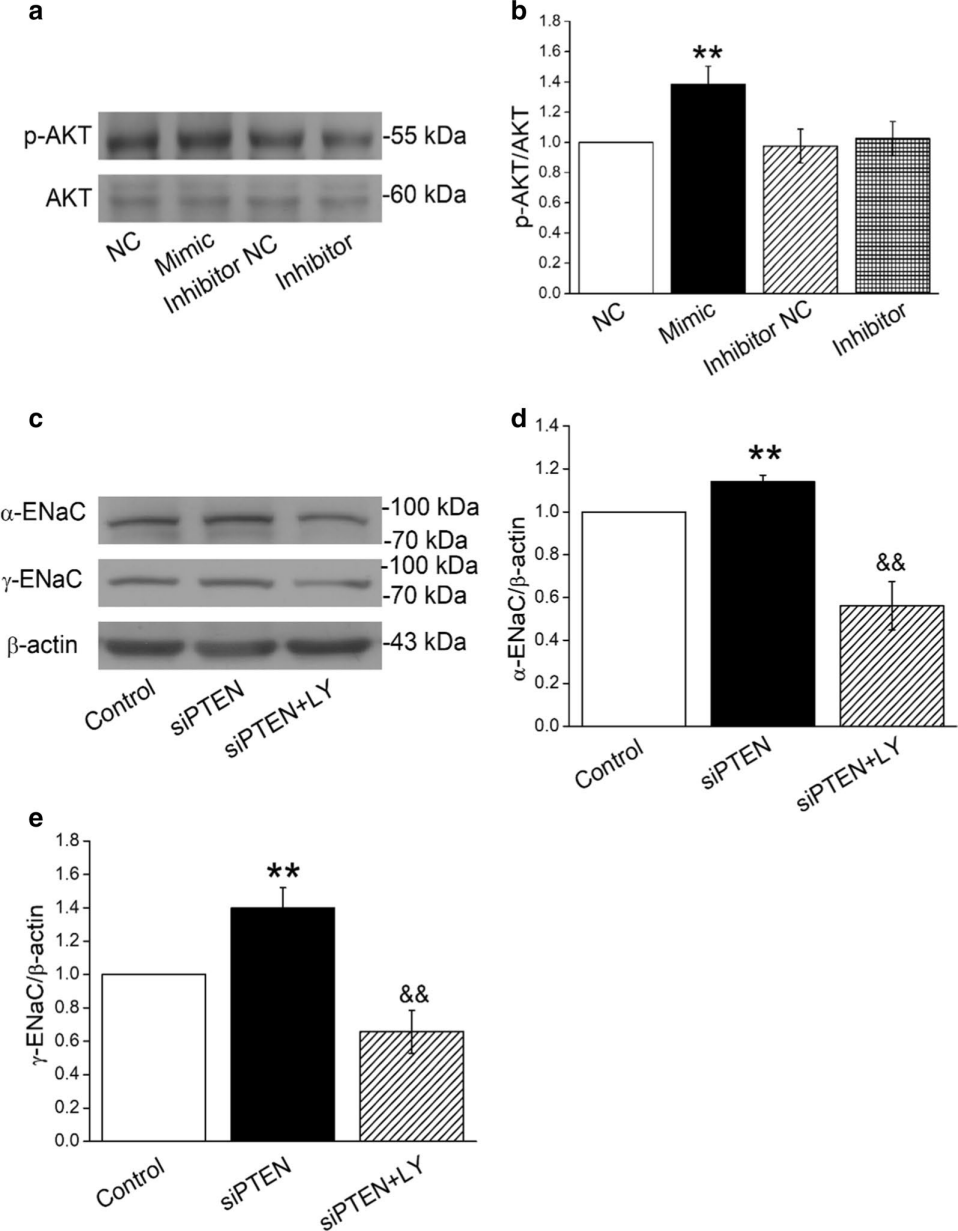
MiR-130b and PTEN regulate α/γ-ENaC through PI3K/AKT pathway in AT2 cells. **a** Representative Western blot bands of p-AKT/AKT protein expression in AT2 cells that were transfected with miR-130b mimic/inhibitor. **c** Representative Western blot bands of α/γ-ENaC protein expression in AT2 cells that were treated with 10 µM PI3K/AKT inhibitor (LY294002) and/or siPTEN for 30 min. **b**, **d** and **e** Graphical representation of data obtained from Western blot assays for p-AKT/AKT and α/γ-ENaC. Bands were quantified using gray analysis (p-AKT/AKT, α-ENaC/β-actin, and γ-ENaC/β-actin). **P < 0.01, compared with Control or miR-130b mimic negative control (NC); ^&&^*P* < 0.01, compared with siPTEN group, n = 4

To test the PI3K/AKT pathway involved in the mechanism of miR-130b-mediated ENaC regulation, we measured the p-AKT/AKT protein expression in AT2 cells that were transfected with miR-130b mimic and inhibitor, respectively. As shown in Fig. 7a, b, miR-130b could increase the p-AKT/AKT protein expression, while inhibition of miR-130b nearly had no effects on the phosphorylation of AKT.

To future confirm the PI3K/AKT as a downstream pathway of PTEN in the miR-130b regulating ENaC, we treated AT2 cells with 10 µM PI3K/AKT inhibitor (LY294002) and/or siPTEN for 30 min, respectively. As shown in Fig. 7c–e, both α and γ-ENaC protein expressions were increased after AT2 cells were transfected with siPTEN, which could be reversed by the co-administration of LY294002 (siPTEN + LY). The above data demonstrate that PTEN/PI3K/AKT pathway may be involved in the miR-130b regulation of α/γ-ENaC in AT2 cells.

## Discussion

ALI is a serious lung disease characterized by sustained edema and lung tissue injury [31], which leads to respiratory failure, with high morbidity and mortality [32]. The discovery of novel and effective therapeutic targets for ALI is of great significance for improving the life quality of patients, and MSCs may be a promising alternative for ALI treatment, as a cell-based therapy [33]. Research data have shown that MSCs reversed ALI-induced decrease of AFC, foremostly contributed by ENaC-mediated fluid transport [34, 35]. In this study, we first found that the expression of ENaC protein and mRNA in AT2 cells increased after the administration of BMSCs, contrary to the effects of LPS alone. To test the effects of BMSCs on ion transport function, *Isc* was measured by ussing chamber assay and we revealed that BMSCs promoted ASI associated with ENaC activity in intact H441 monolayers.

MiRNAs secreted by MSCs are involved in many pulmonary diseases, including ALI, which are especially important in lung homeostasis and development [36]. By performing microarray hybridization, Chen tested the RNAs for the presence of miRNAs in MSC, which expressed at least 151 miRNAs including some passenger miRNA sequences [37]. We have previously testified several miRNAs, including miR-124-5p, miR-130b, and so on. In our recently published paper, we investigated miR-124-5p, which existed in MSCs-conditioned medium, and found that the miR-124-5p was involved in the regulation of MSCs-conditioned medium in LPS-induced ALI by targeting α-ENaC [24]. Of note, miR-130b has been reported to exert key roles in various inflammatory diseases, which suppress IL-6 and TNF-α in mitigating LPS-induced vascular inflammation [18, 19], whereas its role in ALI is seldom studied. The fluorescently labeled miRNA could be observed in co-cultured AT2 cells after we delivered it to the BMSCs, which provided a direct evidence that miR-130b in BMSCs had been transferred into epithelial cells they were co-cultured with. We found that miR-130b was lowly expressed in LPS-treated AT2 cells, and BMSCs could increase the expression of miR-130b. Besides, transfection of miR-130b in either AT2 cells directly or BMSCs indirectly could increase α/γ-ENaC protein expression, indicating miR-130b a key factor in BMSC upregulation of ENaC.

**Fig. 8:**
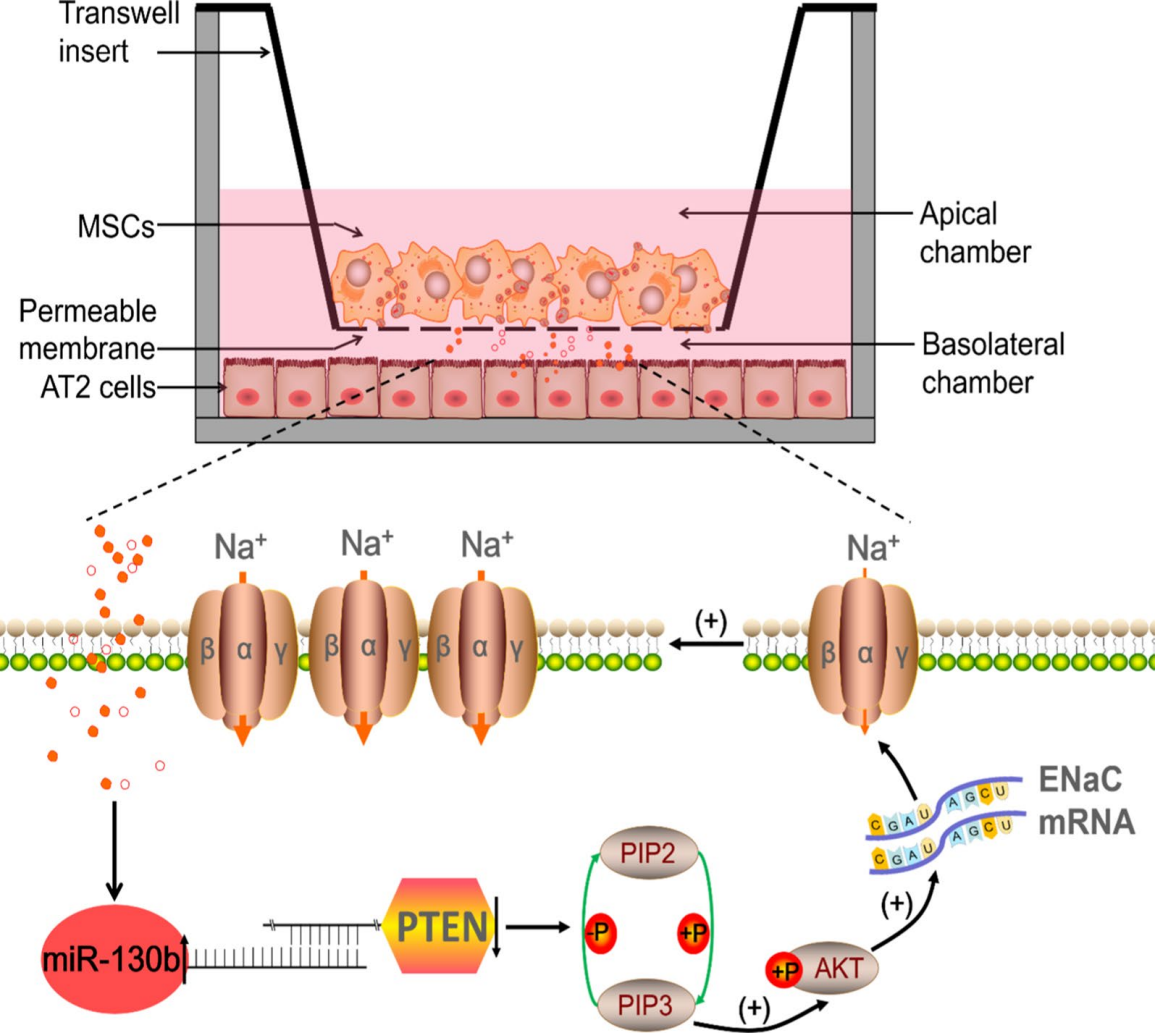
A schematic diagram highlighting the regulation of ENaC by miR-130b involved in MSCs co-cultured AT2 cells. MiR-130b secreted from MSCs cultured in transwell inserts can bind directly to PTEN, which down-regulates PTEN and inhibits the PIP3 dephosphorylation to PIP2. Increased PIP3 can activate AKT, and increase the mRNA/protein expression and function of ENaC in alveolar epithelial cells. *MSCs* mesenchymal stem cells, *ENaC* epithelial sodium channel, *PTEN* phosphatase and tensin homolog deleted on chromosome ten, *PIP2* phosphatidylinositol (4,5)-bisphosphate, *PIP3* phosphatidylinositol (3,4,5)-trisphosphate, *AKT* protein kinase B, *AT2* alveolar type 2 epithelial cells

PTEN as a potential miR-130b target was predicted according to the relative websites, which exerted protective effects in animal models of ALI [38]. An innovative identification in our study revealed that the expression of PTEN in AT2 cells decreased after the administration of BMSCs, further studies showed that overexpressed miR-130b could reduce PTEN protein expression and vice versa, revealing that PTEN might be a target of miR-130b modulating ENaC, which was identified by our dual luciferase target gene assay. Powerful evidence for the targeting effect of PTEN mediating miR-130b regulated ENaC was explored by PTEN siRNA, and we found that LPS-induced α/γ-ENaC reduction were abrogated by knocking down PTEN, while transfection with PTEN-siRNA and miR-130b exerted coincident effects on the expression of α/γ-ENaC in AT2 cells after LPS administration. The degradation of PTEN by miR-130b may activate PI3K/AKT, which inhibits ENaC degradation from plasma membrane through Nedd4-2 [10, 39]. Our results showed miR-130b could increase the p-AKT/AKT protein expression, supporting that PI3K/AKT pathway may be an intermediate of the ENaC regulation by miR-130b. PI3K/AKT inhibitor LY294002 could reverse the enhancement of α/γ-ENaC protein expressions transfected with siPTEN in AT2 cells, implying that PTEN/PI3K/AKT may be involved in the miR-130b regulation of α/γ-ENaC in AT2 cells (Fig. 8), consistent with the similar pathway in miR-130b-mediated cellular protection in several labs [21, 40, 41].

## Conclusions

MiR-130b may enhance ENaC and involve in the BMSCs-based therapy of ALI by targeting PTEN and activating PI3K/AKT pathway in LPS-treated AT2 cells, which represents a promising direction for therapeutic strategy of ALI.

## Supplementary information

**Additional file 1: Figure S1.** The screening results from the websites. The left of this screenshot showed the corresponding scores of miR-130b binding sites, and the right were the two typical miR-130b/PTEN alignments.

**Additional file 2: Figure S2.** Knockdown identification of *PTEN* gene. (A) Representative Western blot measurement of PTEN transfected with PTEN-siRNA (siPTEN). (B) Graphical representation of data obtained from Western blot assays. Bands were quantified using gray analysis (PTEN/β-actin). ^**^P < 0.01, compared with negative control (NC), n = 4.

## Data Availability

The datasets used and analysed during the current study are available from the corresponding author on reasonable request.

## Abbreviations

ALI: Acute lung injury
ENaC: Epithelial sodium channel
AT2: Alveolar type 2 epithelial
MSCs: Mesenchymal stem cells
BMSCs: Bone marrow-derived MSCs
IL: Interleukin
PTEN: Phosphatase and tensin homolog deleted on chromosome ten
PI3K/AKT: Phosphatidylinositol 3-kinase/protein kinase B
ARDS: Acute respiratory distress syndrome
LPS: Lipopolysaccharide
AFC: Alveolar fluid clearance
MiRs: MicroRNAs
Isc: Short-circuit current
