# Supplementary figures and images for: Bone marrow mesenchymal stem cells derived miRNA-130b enhances epithelial sodium channel by targeting PTEN
Source: Respir Res. 2020 Dec 11;21:329. doi: 10.1186/s12931-020-01595-7 (PMC7731743; doi:10.1186/s12931-020-01595-7)

Additional Figure S1


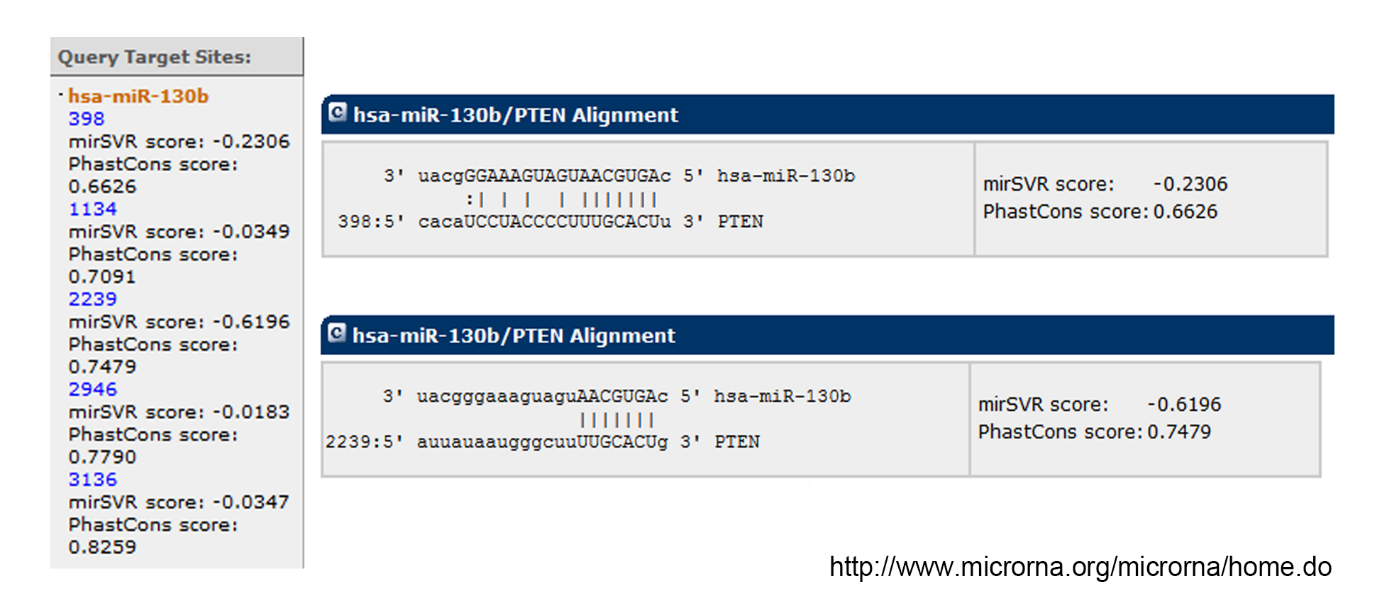

Supplement: Supplementary file 1 — Additional file 1: Figure S1. The screening results from the websites. The left of this screenshot showed the corresponding scores of miR-130b binding sites, and the right were the two typical miR-130b/PTEN alignments. [file 12931_2020_1595_MOESM1_ESM.docx]

Additional Figure S2


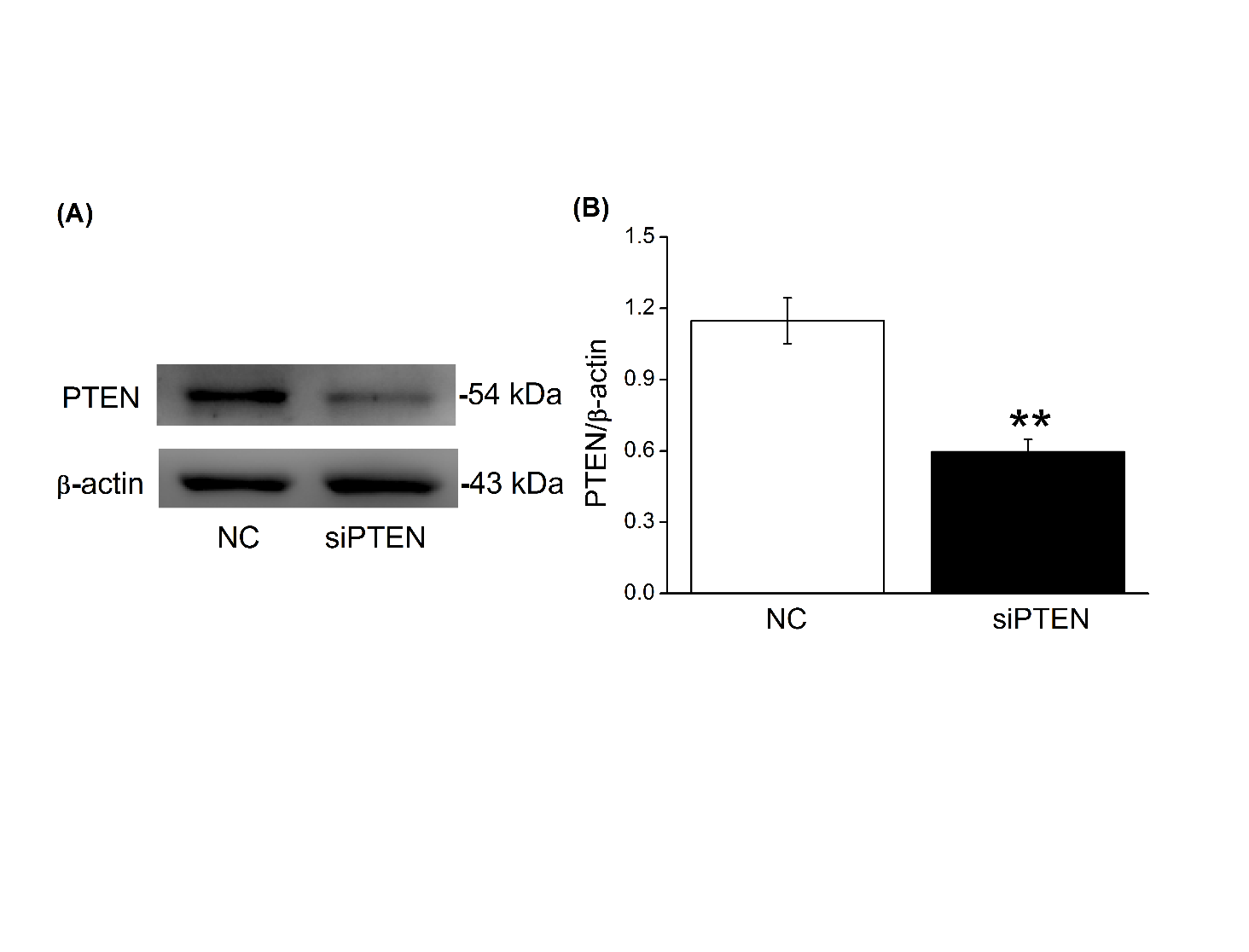

Supplement: Supplementary file 2 — Additional file 2: Figure S2. Knockdown identification of PTEN gene. (A) Representative Western blot measurement of PTEN transfected with PTEN-siRNA (siPTEN). (B) Graphical representation of data obtained from Western blot assays. Bands were quantified using gray analysis (PTEN/β-actin). **P < 0.01, compared with negative control (NC), n = 4. [file 12931_2020_1595_MOESM2_ESM.docx]
